# Cash flow management and its effect on firm performance: Empirical evidence on non-financial firms of China

**DOI:** 10.1371/journal.pone.0287135

**Published:** 2023-06-20

**Authors:** Fahmida Laghari, Farhan Ahmed, María de las Nieves López García

**Affiliations:** 1 School of Accounting, Xijing University, Xi’an City, Shaanxi Province, People’s Republic of China; 2 Department of Economics and Management Sciences, NED University of Engineering & Technology, Karachi City, Pakistan; 3 Department of Business and Economics, University of Almeria, Almería, Spain; Universite de Kairouan, TUNISIA

## Abstract

The main purpose of this research is to investigate the impact of changes in cash flow measures and metrics on firm financial performance. The study uses generalized estimating equations (GEEs) methodology to analyze longitudinal data for sample of 20288 listed Chinese non-financial firms from the period 2018:q2-2020:q1. The main advantage of GEEs method over other estimation techniques is its ability to robustly estimate the variances of regression coefficients for data samples that display high correlation between repeated measurements. The findings of study show that the decline in cash flow measures and metrics bring significant positive improvements in the financial performance of firms. The empirical evidence suggests that performance improvement levers (i.e. cash flow measures and metrics) are more pronounced in low leverage firms, suggesting that changes in cash flow measures and metrics bring more positive changes in low leverage firms’ financial performance relatively to high leveraged firms. The results hold after mitigating endogeneity based on dynamic panel system generalized method of moments (GMM) and sensitivity analysis considering the robustness of main findings. The paper makes significant contribution to the literature related to cash flow management and working capital management. Since, this paper is among few to empirically study, how cash flow measures and metrics are related to firm performance from dynamic stand point especially from the context of Chinese non-financial firms.

## Introduction

Firms’ efficient cash flow management is significant tool to enhance financial performance [1, 2]. Exercising proper management of cash flow is vital to the persistence of business [3]. Cash flow management is primarily concerned with identifying effective policies that balance customer satisfaction and service costs [4]. Firms manage efficiently of cash flows via working capital by balancing liquidity and profitability [5–7]. Working capital management, which is the main source of firm cash flow has significant importance in the context of China, where firms are restricted with limited access to external capital markets. In order to fulfill their cash flow needs firms heavily depend on internal funds, short-term bank loans, and trade credit in order to finance their undertakings [5]. For such firms’ working capital plays the role of additional source of finance. Consistent with this view, KPMG China [8] declared that effective management of working capital has played a vital role to alleviate the effects of recent financial crisis. Additionally, in recent times the remarkable growth of China roots to Chinese private firms’ effective management of working capital in general and their accounts receivables in particular [9]. Therefore, efficient management of working capital is an avenue that highly influence firm profitability [10–12], liquidity [7, 13], and value. Since corporates cash flow management policies settle working capital by account receivables, inventories and accounts payables. Hence, existing theories of working capital management support the view that by cash flow manipulation firms can enhance liquidity and competitive positioning [6, 14, 15]. Therefore, firms manipulate cash flows through its measures, as by way speedy recovery of accounts receivables, reducing inventories, and delaying accounts payables [16]. Hence, the first research question is whether changes in cash flow measures are the tools that could bring positive changes in firm financial performance.

From the accounting perspective, liquidity management evaluates firm’s competence to cover obligations with cash flows [17, 18], as uncertainty about cash flow increases the risk of collapse in most regions, industries, and other subsamples [19]. There are two extents: static or dynamic views, through which corporate liquidity can be inspected. The balance sheet data at some given point of time is a basis for static view. This comprises of traditional ratios such as, current ratios and quick ratios, in order to evaluate firms ability to fulfill its obligations through assets liquidation [20]. The static approach is commonly used to measure corporate liquidity, however, authors also declare that financial ratio’s static nature put off their capability to effectively measure liquidity [21, 22]. The dynamic view is to be utilized to capture the firms’ ongoing liquidity from firm operations [16, 21]. Therefore as a dynamic measure, the cash conversion cycle (CCC) is used by authors to measure liquidity in empirical studies of corporate performance [23]. For instance; Zeidan and Shapir [24] and Amponsah-Kwatiah and Asiamah [25] find that reducing the CCC by not affecting the sales and operating margin increases share price, profits and free cash flow to equity. Accordingly, Farris and Hutchison [20] find that shorter cash conversion cycle leads to higher present value of net cash flows generated by asset which contribute to higher firm value. Moreover, Kroes and Manikas [1] used operating cash cycle as a measure for cash flow metrics, which combines accounts receivables and firm inventory. As explained by Churchill and Mullins [26] that all other things being constant shorter the operating cash cycle faster the company can reassign its cash and can have growth from its internal resources. The second research question therefore is that whether changes in cash flow metrics bring positive improvements in firm financial performance.

Study uses CSMAR database of Chinese listed companies from the period 2018:q2-2020:q1. In the study, measure of firm performance is Tobin’s-q. Study uses three cash flow measures; accounts receivables turning days, inventory turning days and accounts payable turning days, and cash conversion cycle and operating cash cycle as measure for cash flow metrics. Consistent with the prediction, study finds that changes in cash flow measures and metrics bring positive improvements in firm financial performance. In particular decline in cash flow measures (ARTD, ITD, and APTD) to one unit would increase firm performance approximately 6.8%, 0.03%, and 7.2%; respectively. Additionally, one unit decline in cash conversion cycle would increase firm performance approximately 3.8%. Furthermore, study uses GMM estimator to alleviate the endogeneity and observe that the main estimation results still hold. In addition, study also employs a sensitivity analysis specifications to better isolate the impact of changes in cash flow measures and metrics on firm financial performance in previous period and observe that negative association is still sustained.

The sizable number of listed firms in China enable the study to divide sample into two subsamples: firms in high leverage industry and firms in low leverage industry. The study repeats the test on these two subsamples. Significant and negative association between cash flow measures, metrics and firm financial performance is still sustained. Moreover, the results of differential coefficients across two sub samples via seemingly unrelated regression (SUR) systems indicated that cash flow measures and metrics are more pronounced in low debt industries.

The paper makes significant contribution to the literature related to cash flow management and working capital management. First, this paper is among few to empirically study, how cash flow measures and metrics are related to firm performance from dynamic stand point especially in the Chinese context. The study sheds light on the role of cash flow management in improving the firm’s financial performance. Second, extant researches on cash flow management focus on the manufacturing industries. Unlike others this paper investigates the relation between cash flow measures, metrics and firm performance in the context of whole Chinese market, which is essential to know how these performance levers contribute to financial performance of other industries also. Third, results highlight the role of cash flow management in improving financial performance by taking firms’ leverage into consideration and declare that low leveraged industries are better off in terms of influence of changes in cash flow measures and metrics on firm performance. Fourth, the present paper uses generalized estimating equations (GEEs) Zeger and Liang [27] technique which is robust to estimate variances of regression coefficients for data samples that display high correlation between repeated measurements. Finally, to ensure robustness of findings the study uses sensitivity analysis, and in order to control for the potential issue of endogeneity the present study also uses generalized method of moments (GMM) following statistical procedures of Arellano and Bover [28] and Blundell and Bond [29].

The remainder of the paper is organized as follows. Section two discusses the role of cash flow management in China. Section three discusses the relevant literature, theoretical framework and development of hypotheses. Section four presents the data and variables of the study. Section five reports the methodology, empirical results and discussions. Section six concludes the paper.

## Cash flow management in China

The economy of China has undergone a massive economic growth rates followed by high rates of fixed investment in the past three decades [5, 30]. This growth miracle is outcome of highly productive firms and their ability to accrue significant cash flows [31], despite inadequate financial system. Moreover, although Chinese economy has seen fast growth and development in the past two decades but still the legal environment in China cannot be regarded as conducive [32, 33]. As, in the credit market of China government plays a decisive role in credit distribution [34, 35], and mostly the credit is granted to companies owned by state or closely held firms [34, 36]. The Chinese firms have restricted admittance to the long-standing funds marketplace [37], therefore, companies held private or non-SOE find difficulty to access credit from financial market relatively to state owned firms. Although by the 1998 leading Chinese banks were authorized to lend credit to privately held firms but still these firms face troublesome to get external finance comparatively to state owned firms [32]. The prior literature also indorses this and states that with the presence of regulatory discrimination amid privately held and state owned firms, the privately held firms to the extent are often the subject of state predation [38, 39].

Given country’s poor financial system, firms in China have managed their growth rates from their internal resources. Working capital management from where firms manage cash flows is the source of financing of the growth by Chinese firms. Accordingly, Ding *et al*. [5] mentioned that in their sample of Chinese firms about 66.6% dataset were characterized by a large average ratio of working capital to fixed capital, as it is a source and use of short term credit. Additionally, Dewing [40] termed working capital as one of the vital elements of the firm along with fixed capital. Moreover, Ding *et al*. [5] conclude that in the presence of financial constraints and cash flow shocks still Chinese firms can manage high fixed investment levels which correspond more to working capital than fixed capital. They further state that this all roots to the efficient management of working capital that Chinese firms use in order to mitigate liquidity constraints.

## Literature review, theoretical background and hypothesis development

### Literature review and theoretical background

Corporate finance theory states that the main goal of a corporation is to maximize shareholder wealth [41]. Neoclassical capital theory is based on the proposition put forward by Irving Fisher [42] that individual consumption decisions can be separated from investment decisions. Fisher’s separation theorem holds true in perfect capital markets, where companies and investors can lend and borrow on the same terms without incurring transaction costs. In such a world, the choice to change income streams by lending and borrowing to meet preferences of consumption means that investors rank income streams according to their present value. Therefore, the value of the company is maximized by choosing the set of investments that generate the largest net present value over returns. When the company pays cash dividends with capital reserves, cash dividends can be maintained at a certain level, and when the ratio of capital reserves to cash dividends is high, accrual income management is low [43]. Since Gitman’s [44] seminal work, in which he introduced the concept of cash circulation as a means of managing corporate working capital and its impact on firm liquidity. Richards and Laughlin [16] then transformed the cash cycle concept into the Cash Conversion Cycle (CCC) theory for analyzing the working capital management efficiency of firms. CCC theory holds that effective working capital management (i.e., shorter cash conversion cycles) will increase a company’s liquidity, all else being equal. Signal theory can illustrate how a company can provide excellent signals to users of financial and non-financial statements [45]. In addition, this theory can also be used as a reference for investors to see how good or bad a company is as an investment fund. This theory explains the relationship between working capital turnover and profitability.

The trade-off theory in capital structure is a balance of benefits and sacrifices that may occur due to the use of debt [46]. The higher the amount a company spends on financing its debt, the greater the risk that they will face financial hardship due to excessive fixed interest payments to debt holders each year and uncertain net income. Higher cash flow uncertainty leads to an increased risk of business collapse [19]. Companies with high levels of leverage should keep their liquid assets high, as leverage increases the likelihood of financial distress. This theory is used to explain the relationship between leverage and profitability. Pecking order theory explains that companies with high liquidity levels will use more debt funds than companies with low liquidity levels [47]. Liquidity measures a company’s ability to meet its cash needs to pay short-term debts and fund day-to-day operations as working capital. The better the company’s current ratio, the more the company will gain the trust of creditors so that creditors will not hesitate to lend the company funds used to increase capital, which will benefit the company.

Prevailing working capital management theories argue that firms can improve their competitive position by manipulating cash flow to improve liquidity [14, 15, 20, 48–50]. In addition, the company’s ability to convert materials into cash from sales reflects the company’s ability to effectively generate returns from investments [51]. It’s better to combine investment spending with cash flow from ongoing operations than to measure and report both discretely [52]. Three factors directly affect the company’s access to cash: (i) the company’s inability to obtain cash receivables while waiting for the customer to pay for the delivered goods; (ii) the company is unable to obtain cash receivables; (iii) the company is unable to obtain cash receivables. (ii) Cash invested in goods is tied up and unavailable and the goods are inventoried; and (iii) cash may be made to the company if it chooses to delay payment to suppliers for goods or services provided [16]. While a company’s cash payments and collections are typically managed by the company’s finance department, the three factors that affect cash flow are primarily manipulated by operational decisions [53].

In the literature, the prevailing view is that the presence of liquidity is not always good for the company and its performance, because sometimes liquidity can be overinvested. Since emerging markets are characterized by imperfect markets, companies maintain internal resources in the form of liquidity to meet their obligations. As in emerging markets, financial markets are inefficient in allocating resources and releasing financial constraints, resulting in underinvestment by financially constrained companies [54]. In addition, access to capital markets, external financing costs, and availability of internal financing are financial factors on which a company’s investments rely [55]. Alternatively, the pecking order theory [56] argues that due to information asymmetry, companies adopt a hierarchical order of financing preferences, so internal financing takes precedence over external financing. A study by Zimon and Tarighi [7] argue that businesses must use the right working capital strategy to achieve sustainable growth as it optimizes operating costs and maintains financial liquidity. Moreover, asset acquirements affect a company’s output and performance [57].

The existing literature provides different evidence of the impact of working capital management on firm performance. A study by Sharma and Kumar [58] examine the relationship between working capital management and corporate performance in Indian firms. Considering a sample of 263 listed companies during the period 2000–2008, they found that CCC had a positive impact on ROA. Similarly, of the 52 Jordanian listed companies in the period 2000–2008, Abuzayed [11] found a positive impact of CCC on total operating profit and Tobin’s-Q. Similar findings have been reported by companies in China [59], the Czech Republic [60], Ghana [25], Indonesia [6], Spain [61], and Visegrad Group countries [62]. In contrast, few studies reported an inverse correlation between CCC and firm performance in India [63], Malaysia [2], and Vietnam [64]. A negative correlation indicates that a higher CCC leads to lower company performance. A study by Afrifa et al. [65] did not find any significant relationship between CCC and firm performance. The findings of the relationship between NWC and company performance are not much different from CCC. Companies in European countries [66], and the United Kingdom [67] reported positive correlations, and those in Poland reported negative correlations [68]. Although previous operations management studies have explored the relationship between working capital and firm performance, the results of these studies remain inconclusive, and the study has found positive, curved, and even insignificant relationships. This is mainly since accidental factors make this relationship both complex and special. Therefore, to enhance the beneficial impact of working capital and cash flow on corporate performance, companies must make appropriate investments to promote more objective, informed, and business-specific working capital and cash flow management choices [69]. Collectively, these mixed pieces of evidence provide sufficient motivation for this study to develop hypotheses based on positive and negative relationships.

### The cash flow measures and firm financial performance

The firms’ trade where merchandise sold on credit instead of calling for instantaneous cash imbursement, such transaction generate accounts receivables [70]. Accounts receivable directly affect the liquidity of the enterprise, and thus the efficiency of the enterprise [71]. From the stands of a seller, the investment in accounts receivables is a substantial component in the firm’s balance sheet. Firms’ progressive approach towards significant investment in accounts receivables with respect to choice of policies for credit management contributes significantly to enhance firm value [72]. Firms can utilize cash received from customers by investing in activities which contribute to enhance sales [1]. Firms can improve liquidity position with capability to collect overheads from customers for supplied goods and services rendered in a timely manner [17]. However, credit sales is instrumental to increase sales opportunities for firms but may also increase collection risk which can lead to cash flow stresses even to healthy sales growth companies [73]. Firms offer sales discounts which may not increase sales but may increase payments by customers and improve firms’ cash flow, reduce uncertainty of future cash flows, reduce risk and required rate of return [74].

**Fig 1:**
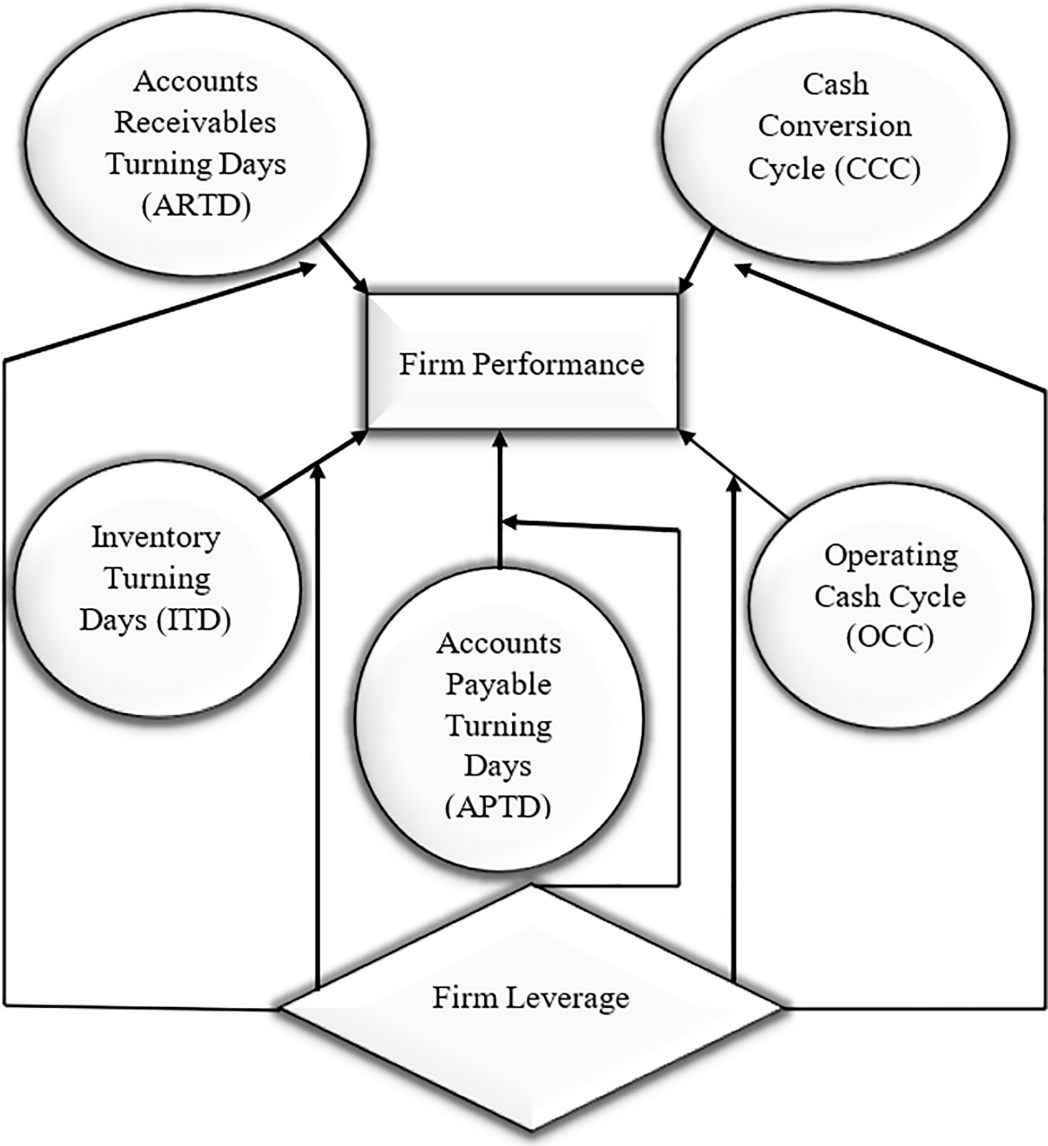
Conceptual model of the study.

Literature suggests that firm performance increases with shorter period of day’s sales outstanding [15, 20, 26]. Accordingly, Deloof [75] by working on Belgians firms find negative relationship between number of days accounts receivables and gross operating income. However, models of trade credit (such as; Emery, [21]) endorse that higher profits also lead to more accounts receivables as firms with higher profits are rich in cash to lend to customers. In a study by García-Teruel and Martinez- Solano [76] suggest that managers of firms with fewer external financial resources available generally dependent on short term finance and particularly on trade credit that can create value by shortening the days sales outstanding. Furthermore, Gill *et al*. [10] declare that firm can create value and increase profitability by reducing the credit period given to customers. Kroes and Manikas [1] analyzed manufacturing firms and suggested that decline in days of sales outstanding relates to improvements in firm financial performance and persists to several quarters. According to Moran [77] suppliers happily offer reasonable sales discounts for early payments which improve their cash flow position, locks the receivables, remove the bad debt risk at early stage, and reduce their day’s sales outstanding significantly which ultimately improve their working capital position. Fig 1 depicts this relationship. In consistent with discussion the following hypothesis is proposed:

**H1a**: A decrease (increase) in the duration of accounts receivables turning days increases (decreases) firm financial performance.

The research has mixed views whether reduction in inventory is beneficial to firm performance or increase in inventory leads to increased performance. Despite high cash flow, inventory level management has been neglected [78]. In this regard literature has evidenced three themes of relationships: positive relationship, negative relationship or no relationship, and inclusion of moderators and mediators to the relationship of number of day’s inventory and firm performance [79]. However, the inventory management revolutionized after the launch of lean system with familiarizing just-in-time inventory philosophy by Japanese companies [80, 81]. Afterwards, research related to inventory management evidenced that firms which adopted lean system not only improved customer satisfaction but also attained greater level of asset employment that ultimately leads to higher organizational growth, profitability, and market share [82, 83]. Moreover, in a JIT context firms experience positive effects on organizational performance due to reduced inventory, and reduction in inventory significantly improves three performance measures such as: profits, firms return on sales, and return on investments [84]. Additionally, Fullerton and McWatters [85] found positive influence of reduced inventory on organizational performance which corresponds to JIT context.

However, generally literature considers that better inventory performance such as: higher inventory turns or decreased level of inventory is normally attributed to better firm financial performance [86]. Moreover, it is a mutual consent by researchers that high level of inventory also signifies demand and supply misalliance and often related to poor operational performance [87, 88]. In a study by Elsayed and Wahba [79] indicated that there is influence of organizational life cycle on the relationship of inventory and organizational performance. Their results indicated that at initial stage though ratio of inventory to sales negatively affects organizational performance, but it put forth significant and positive coefficient on organizational performance at the revival phase or rapid growth phase. Additionally, literature has documented negative influence of reduced inventory on performance. In a study by Obermaier and Donhauser [89] evidenced that lowest level of inventory leads to poor organizational performance and suggest that moving towards zero inventory case is not always favorable. Fig 1 depicts this relationship. Accordingly the hypothesis is proposed as follows:

**H1b**: A decrease (increase) in the duration of inventory turning days increases (decreases) firm financial performance.

According to Deloof [75] payment delays to suppliers are beneficial to assess the quality of product bought, and can serve as a low-cost and flexible basis of financing for the firm. On the contrary, delaying payments to suppliers may also prove to be costly affair if firm misses the discount for early payments offered [90], hence firms by reducing days payable outstanding (DPO) likely to enhance firm financial performance [76]. In line with this, Soenen [22] states that firms try to collect cash inflows as quickly as possible and delay outflows to possible length. Payment delays enable firms to hold cash for longer duration which ultimately increases firms’ liquidity [50]. As discussed by Farris and Hutchsion [20] that firms can improve cash to cash cycle by extending the average accounts payable along with inventory and get interest free financing. A study by Sandoval *et al*. [91] speculate that investors are more sensitive to accruals of long-term operating assets than to accruals of long-term operating liabilities because the former is more associated with recurring profits than the latter. Moreover, Fawcett *et al*. [92] indorsed that by extending the duration of accounts payable cycle companies can improve their cash to cash cycle. However, longer payment cycles not only harm relationship with suppliers, but may also lead to lower level of services from suppliers [93].

As discussed by Raghavan and Mishra [94] firms may be reluctant to produce or order at optimal point followed by cash restraints for fast growing firms where money plays the role of catalyst when demand is significantly high but firms are financially restricted to order less and this situation may mark the harmful effects over the performance of whole supply chain at least on temporary basis until restored. Hence, this situation is favoring that firms encourage and motivate their customers for quicker payments in order to increase cash to cash cycles [92]. Fig 1 depicts this relationship. Accordingly based on discussion hypothesis is proposed as follows:

**H1c**: A decrease (increase) in the duration of accounts payable turning days’ increases (decreases) firm financial performance.

### The cash flow metrics and firm financial performance

As shown by Richards and Laughlin [16] that firms should collect inflows as quickly as possible and postpone cash outflows as long as possible which is a general view based on the concepts of operating cash cycle (OCC) and cash conversion cycle (CCC). This shows that firms by reducing CCC cycle can make internal operation more efficient that ensures the availability of net cash flows, which in turn depicts a more liquid situation of the firm, or vice versa [25]. They further said that cash conversion cycle (CCC) is based on accrual accounting and linked to firm valuation. Baños-Caballero *et al*. [95] suggested that however, higher level of CCC increases firm sales and ultimately profitability, but may have opportunity cost because firms must forgo other potential investments in order to maintain that level. On the contrary, longer duration of CCC may hinder firms to be profitable because this is how firms’ duration of average accounts receivables and inventory turnover increase which may lead firms towards decline in profitability [96]. Therefore, cash conversion cycle (CCC) can be reduced by shortening accounts receivables period and inventory turnover with prolonged supplier credit terms which ultimately enable firms to experience higher profitability [97, 98]. A shorter duration of CCC helps managers to reduce some unproductive assets’ holdings such as; marketable securities and cash [23]. Because with low level of CCC firms can conserve the debt capacity of firm which enable to borrow less short term assets in order to fulfill liquidity. Therefore, shorter CCC is beneficial for firms that not only corresponds to higher present value of net cash flows from firm assets but also corresponds to better firm performance [60, 62].

Operating cash cycle is a time duration where firm’s cash is engaged in working capital prior cash recovery when customers make payments for sold goods and services rendered [16, 26]. Literature endorses that shorter the operating cash cycle better the firm liquidity and financial performance because companies can quickly reassign cash and cultivate from internal sources [16]. In a study by Kroes and Manikas [1] find that there is significant negative relationship between changes in OCC with changes in firm financial performance. They further suggested that OCC can be taken by managers as a metric to monitor firm performance and can be used as lever to manipulate in order to improve firm performance. A study by Farshadfar and Monem [99] also found that when the company’s operating cash cycle is shorter and the company is small, the cash flow component improves earnings forecasting power better than the accrual component. Moreover, Nobanee and Al Hajjar [100] recommend the optimum operating cycle as a more accurate and complete working capital management measure to maximize the company’s sales, profitability, and market value. Fig 1 depicts this relationship. Hence, based on above discussion the proposed hypotheses are:

**H2a**: A decrease (increase) in cash conversion cycle increases (decreases) firm financial performance.
**H2b**: A decrease (increase) in operating cash cycle increases (decreases) firm financial performance.

## Data and variables

### Samples selection

The data used in this study is taken from China Stock Market and Accounting Research (CSMAR) database. The study includes quarterly panel data of non-financial firms with A-shares listed on Shanghai Stock Exchange (SHSE) and Shenzhen Stock Exchange (SZSE). The data comprises on eight quarters ranging from 2018:q2-2020:q1, and four lag effects are included that make data up to twelve quarters. The use of quarterly data ensures greater granularity in the findings of the study as prior studies have mainly used annual data, therefore, this study uses two years plus one year of lagged data which offers exclusively a robust sample period that is instrumental to effective inference [1]. The main benefit of this method of examining quarterly changes within a company is that the company cannot have any missing data items throughout the sample period. Because any missing data will lead to design errors and imbalance panel data. Therefore, this problem led to now selection of a 12-quarter observation frame (two years plus one year of lagging data) because it delivers a reliable sample period from which effective conclusions can be prepared. Moreover, the data is further refined and maintained from unobserved factors, unbalanced panels, and calculation biases. Moreover, deleted firm-year observation with missing values; excluded all financial firms; as their operating, investing, and financing activities are different from non-final firms [75, 101], eliminated firms with traded period less than one year, and excluded all firms with less than zero equity. The data is further winsorized up to one percent tail in order to mitigate potential influence of outliers [76]. Additionally, the firms’ data with negative values for instance; sales and fixed assets is also removed [67, 101]. The final sample left with balanced panel of 20288 firm year observations consists of 2536 groups. The change (Δ) in all dependent and independent variables of the study sample represents variable period t measured as difference between value at the end of current quarter and value of the variable at the end of prior quarter divided by value of the variable at the end of prior quarter.

### Dependent variable

The firm’s financial performance is dependent variable in the study and is measured through Tobin’s-q. Tobin’s-q is the ratio of firm’s market value to its assets replacement value and it is widely used indictor for firm performance [1, 102–105]. Tobin’s-q diminishes most of the shortcomings inherent in accounting profitability ratios as accounting practices influence accounting profit ratios and valuation of capital market applicably integrates firm risk and diminishes any distortion presented by tax laws and accounting settlements [106]. Moreover, this variable has preference over other accounting measures (such as; ROA) as an indicator of relative firm performance [107].

### Independent variables

Based on established literature [1, 5, 12, 75, 76] this study has used three cash flow measures and two composite metrics as independent variables. Each one of them is discussed below.

#### Accounts receivables turning days (ARTD)

Accounts receivables turning days (ARTD) are the average number of days required by firms for collecting the payments from customers after the sales is done [76]. Following Ding *et al*. [5] and Tahir and Anuar [12] the accounts receivables turning days (ARTD) is measured as:

$$ARTD=\left(Accounts\;Receivables/Sales\right)*\;365$$ (1)

The increasing days of sales outstanding specifies that firm is not handling its working capital efficiently, because it takes longer duration to collect its payments, which signifies that firm may be short of cash to finance its short term obligations due to the longer duration of cash cycle [5].

#### Inventory turning days (ITD)

Inventory turning days (ITD) indicate that how many times the firm is capable to rotate its inventories into sales in duration of a year. Study following Kroes and Manikas [1] and Tahir and Anuar [12] measures proxy for days of inventory turnover as:

$$ITD=\left(Inventories/cost\;of\;goods\;sold\right)*\;365$$ (2)

A higher ratio of inventory turnover is a good sign for firm as it signifies that firm is not having too many products in idle condition on shelves [5].

#### Accounts payable turning days (APTD)

Accounts payable turning days (APTD) are the average number of days taken by a company for payment to its creditors. Following Deloof [75]; Tahir and Anuar [12] the proxy for APTD is measured as:

$$APTD=\left(Accounts\;payables/Purchases\right)*\;365$$ (3)

A firm with higher days of payable outstanding ratio shows that it takes longer duration to make payments to suppliers which is a sign of poor efficiency of working capital, however longer duration of DPO also signifies that company has good terms with suppliers which is also beneficial [5].

#### Cash conversion cycle (CCC)

The cash conversion cycle (CCC) introduced by Gitman [44] (1974) is a dynamic measure of ongoing liquidity management which combines both balance sheet and income statement data. The CCC is a measure of cash outlays for resources and receipt of cash from the sales of a product. Following Deloof [75], Kroes and Manikas [1], and Yazdanfar and Öhman [96] the proxy for CCC is measured as:

$$CCC=\left(Accounts\;receivables\;turning\;days+Inventory\;turning\;days\;–Accounts\;payable\;turning\;days\right)$$ (4)

It is generally considered that lower the CCC cycle better the firm efficiency and able to accomplish its working capital [5]. Additionally, longer duration of CCC shows more time duration between cash outlay and recovery of cash [76].

*Operating Cash Cycle (OCC)*: Operating cash cycle comprises only a subset of cash conversion cycle metric. Following Kroes and Manikas [1] the proxy for operating cash cycle (OCC) is measured as:

$$OCC=\left(Accounts\;receivables\;turning\;days+Inventory\;turning\;days\right)$$ (5)

Operating cash cycle does not take into account the payables, and hence comprises of days where cash is detained as inventory prior receipts of payments from customer [1]. Besides, generally it is considered that firm having shorter OCC is with better liquidity and performance [26].

### Control variables

This study uses firm size and return on assets as control variables. Following Deloof [75] the study uses firm size by taking natural logarithm of quarterly sales. The firm size has significant impact on market value of firms [103, 108]. Study uses quarterly sales instead of total assets as measure for firm size to avoid the potential multicollinearity problem because total asset is denominator for the dependent variable [1]. Following Baños-Caballero *et al*. [95] study controls for return on asset (ROA) which is accounting measure of firms. Return on assets (ROA) is a ratio of earnings before interest and taxes (EBIT) divided by total assets [109].

### Descriptive statistics

Table 1 shows the descriptive statistics of variables of the study. The mean and median value of ARTD is 92.89 and 73.14, respectively. On average, the firms in our sample have relatively higher median value of days of sales outstanding than evidence of Ding *et al*. [5], which shows that Chinese firms take longer to collect their payments from customers. The mean and median value of APTD is 105 and 82.25, respectively. The mean and median value of ITD is 166.18 and 107.13, respectively. On average it shows relatively high inventory turnover in our sample firms which signifies that Chinese firms are quite efficient in inventory management and products are not sitting idle in shelves. The mean and median value of CCC is 150.62 and 115.30, respectively. On average the CCC of Chinese firms is relatively high. However, in a study by Hill *et al*. [101] indicated that higher CCC also signifies higher firm profitability. The mean and median value of OCC is 250.71 and 206.44, respectively. The firm performance (Tobins-q) has a mean and median value of 2.86 and 2.27. The ROA shows mean and median value of 2.46 and 1.67, respectively. On average the size of Chinese firms is 20.79 with median value of 20.71.

**Table 1:** Descriptive statistics.

Variables	Mean	Std. Dev	Q1	Median	Q3	Obs.
ARTD	92.89	74.85	30.41	73.14	136.37	20288
ITD	166.18	151.80	54.70	107.13	220.55	20288
APTD	105.92	81.31	47.67	82.25	136.04	20288
CCC	150.62	128.17	42.58	115.30	235.67	20288
OCC	250.71	159.92	116.05	206.44	369.45	20288
Tobin’s-Q	2.86	2.14	1.22	2.27	3.90	20288
ROA	2.46	2.95	0.44	1.67	4.01	20288
SIZE	20.79	1.40	19.74	20.71	21.76	20288

Note: Please check S1 Appendix for variables descriptions.

The Table 2 reports results for correlation matrix. The correlation coefficient between Tobin’s-Q and CCC is significant and negative at 1 percent level which is consistent to the findings of Afrifa [67]. The correlation between all the measures of cash flows and ROA is significant and negative at 1 percent, consistent with the results of Deloof [75]. Moreover the correlation between ROA and CCC is also significant and negative at 1 percent, similar evidences find by García-Teruel and Martinez-Solano [76] for the sample of Spanish firms. Furthermore, the correlation coefficients among all the variables are significantly lower than 0.80 indicating no sign of multicollinearity [110]. The formal test of variance inflation factor (VIF) for all the independent variables of study were examined to check if there is presence of multicollinearity. The variance inflation factor (VIF) also indicated no multicollinearity among analysis variables with all values below the threshold level of 10 proposed by Field [110], which shows that multicollinearity may not be the case and data is suitable for further analysis.

**Table 2:** Correlation matrix and variance inflation factor (VIF) analysis.

Variables	1	2	3	4	5	6	7	8
1. ARTD	1.00							
2. ITD	0.11***	1.00						
3. APTD	0.47***	0.44***	1.00					
4. CCC	0.11***	0.09***	0.11***	1.00				
5. OCC	0.05***	0.18***	0.13***	0.04***	1.00			
6. Tobin’s-q	0.18***	-0.06***	-0.06***	-0.02***	-0.02***	1.00		
7. ROA	-0.17***	-0.17***	-0.24***	-0.16***	-0.05***	0.26***	1.00	
8. SIZE	-0.08***	-0.04***	-0.03***	-0.03***	-0.37***	-0.12***	0.15***	1.00
VIF	4.86	1.05	4.87	5.36	5.63	(D.V)*	1.06	1.17

Note:

*** denotes statistical significance at the 1% confidence level. Please check S1 Appendix for variables descriptions.

*(D.V): Tobin’s-q is the dependent variable that’s why not shown VIF results.

## Methodology, empirical analysis and discussion

### Effect of cash flow measures on firm financial performance

To investigate the relationship between cash flow measures and firm financial performance, study regresses firm financial performance on cash flow measures and specific control variables. The study followed generalized estimating equations (GEEs) of Zeger and Liang [27] population averaged technique for the analysis. One of the specific advantage of GEEs over other techniques is, its ability to robustly estimate the variances of regression coefficients for data samples that display high correlation between repeated measurements [111, 112]. To model and check the relationship of importance amid the dependent variable and explanatory variables, GEEs practice a function named as link function. Conditional on the dissemination of the dependent variable, many link functions could be stated to line the association amid the forecasted variables and the dependent variable. In the data, the explanatory and explained variables are distributed normally; so, the examines employs the non-transforming function of identity link function Δ(ᴜ_i_)¼ Y_i_Ɓ, where ᴜ_i_¼ È(ýi|Y_i_), and ᵬ signifies the trajectory of coefficients of regression (ᵬ1,…, ᵬn) projected via the GEEs method. The GEEs method evaluates the parameters of model (ᵬ’s) over the iterative process which augments data fit to the research model. Repetitive time-series monetary quantities, for instance; the cash flow mechanisms, display a first order autoregressive link amid time phases [113]. So, the employed association matrix Ř(ẩ) is outlined with the auto-regressive first-order AR(1) condition [27]. The following estimation equations of (GEEs) demonstrates the model of regression for cash flow measures:

$$\begin{array}{l} \Delta Yit=\beta 0+\beta 1\;\left(\Delta X1it\right)+\beta 2\;\left(\Delta X1it-2\right)+\beta 3\;\left(\Delta X1it-2\right)\;+\beta 4\;\left(\Delta X1it-3\right)\;+\beta 5\;\left(\Delta X1it-4\right)+\beta 6\;\left(\Delta X2it\right)+\\ \beta 7\;\left(\Delta X2it-1\right)+\beta 8\;\left(\Delta X2it-2\right)\;+\beta 9\;\left(\Delta X2it-3\right)+\beta 10\;\left(\Delta X2it-4\right)+\beta 11\;\left(\Delta X3it\right)+\beta 12\;\left(\Delta X3it-1\right)+\\ \beta 13\;\left(\Delta X3it-2\right)+\beta 14\;\left(\Delta X3it-3\right)+\beta 15\;\left(\Delta X3it-4\right)+\Sigma \;\beta it\;CONTROLSit+Uit \end{array}$$ (6)

Where ΔY_it_ represents Tobin’s-q for industry i and time t. The ΔX_1it_ is accounts receivable turning days (ΔARTD), and ΔX_1it-1_ to ΔX_1it-4_ are lags for ΔARTD. The ΔX_2it_ is inventory turning days (ΔITD), and ΔX_2it-1_ to ΔX_2it-4_ are lags for ΔITD. The ΔX_3it_ is accounts payable turning days (ΔAPTD), and ΔX_3it-1_ to ΔX_3it-4_ are lags for ΔAPTD. The CONTROLS_it_ represent control variables; Size and ROA. The U_it_ is probabilistic term. Study included four lag effects in Eq 6 for cash flow measures to examine how long the impact of changes in cash flow measures on changes in firm performance persists.

Table 3 provides detailed results of GEEs model’s parameters estimation analysis. The dependent variable is firm performance (Tobin’s-q) in all the models columns 2 through 4. *H1a*, *H1b*, and *H1c* posits that changes in measures of cash flow (ΔARTD, ΔITD, and ΔAPTD) changes firm financial performance. The coefficient of accounts receivable turning days (ΔARTD) in model 1 is -0.0068297, which is statistically significant at 0.1% confidence level in the current quarter. It is consistent with the study’s argument that decline in firms’ days of accounts receivables increases firm financial performance. Similar evidences were found by Shin and Soenen [13], Wilner [114], Deloof [75], and Kroes and Manikas [1]. According to Deloof [75] the negative relationship between days sales outstanding and firm performance suggests that managers can create value for their shareholders by reducing number of day’s accounts receivables to a reasonable minimum.

**Table 3:** Impact of cash flow measures on firm performance.

Ind. Variables	Full Sample Firms Model 1	High leverage Firms Model 2	Low leverage Firms Model 3
Constant	42.6622*** (7.9827)	42.44134*** (7.956666)	41.98058*** (8.12065)
ΔARTD_t_	-0.0068297*** (0.0015414)	-0.0055801*** (0.00086)	-0.0276783* (0.0143908)
ΔARTD_t-1_	9.3e^-07^ *** (5.21e^-08^)	-6.17e-07 (5.31e-07)	0.0439335 (0.0275929)
ΔARTD_t-2_	1.60e^-06^*** (2.01e^-07^)	1.29e-06*** (1.97e-07)	0.0060988*** (0.0007294)
ΔARTD_t-3_	-6.65e^-06^*** (1.71e^-07^)	-6.40e-06*** (2.03e-07)	0.0001188** (0.0000472)
ΔARTD_t-4_	3.79e^-06^*** (1.29e^-07^)	-3.46e-06*** (1.61e-07)	0.0000687 (0.0000466)
ΔITD_t_	-0.0003014*** (0.0000396)	-0.0053208** (0.0019477)	-0.000263*** (0.0000147)
ΔITD_t-1_	0.0002153*** (0.0000144)	-0.0003159 (0.0001956)	0.0000243 (0.0000496)
ΔITD_t-2_	-0.0000342 (0.0000233)	-0.0000788 (0.0000462)	-0.0003622*** (0.0000462)
ΔITD_t-3_	-0.0000492** (0.0000191)	0.0001086 (0.0000731)	-0.0002013** (0.0000693)
ΔITD_t-4_	-7.74e-06 (0.0000128)	0.0001088** (0.0000388)	-0.0062278 (0.003713)
ΔAPTD_t_	-0.0717425*** (0.0145073)	-0.0642792** (0.0227101)	-0.0620844*** (0.0195532)
ΔAPTD_t-1_	0.0003711* (0.0001871)	0.0301199** (0.0108246)	-0.0143467 (0.0091749)
ΔAPTD_t-2_	0.001346*** (0.0002774)	0.0046692* (0.0023402)	-0.0005332 (0.0004199)
ΔAPTD_t-3_	-0.0003244 (0.0003062)	-0.0083566* (0.0040727)	-0.0007271 (0.0005434)
ΔAPTD_t-4_	-0.0001812 (0.0003222)	-0.0060088** (0.0020329)	-0.0000577 (0.0000963)
Size	-2.026229*** (0.3905222)	-2.008665*** (0.389364)	-1.993193*** (0.3962218)
ROA	-0.3742673*** (0.0773292)	-0.3936583*** (0.0791495)	-0.3774285*** (0.0771758)
Wald χ^2^ Statistic	5535.43	71345.48	2693.83
No of groups	2536	2536	2536

Note: Standard errors are in parentheses.

***, **, *; indicate significant at 0.1%, 1%, and 5% respectively.

Please check S1 Appendix for variables descriptions.

The coefficient of inventory turning days (ΔITD) in model 1 is -0.0003014, which is statistically significant at 0.1% confidence level in the current quarter. These results are consistent with the argument given in hypothesis *H1b*. Significant number of studies conclude that low inventory period increases liquidity and firm performance [75, 86, 115, 116]. Moreover, this finding is consistent with literature as firms sound inventory position exhibits better operational and financial performance [117, 118].

The coefficient of accounts payable turning days (ΔAPTD) in model 1 is -0.0717425, which is statistically significant at 0.1% confidence level in the current quarter. These results are consistent with present study’s argument that decline in accounts payable turning days brings positive improvements in firm performance. The findings of results for APTD present strong evidence that when companies reduce their APTD by taking advantage of early discounts payment from suppliers, firms may have a persistent duration of perpetual firm financial performance improvement. As suggested by Moran [77] that firms may be more beneficial by taking advantage of early payment discounts than prolonging the cycle because of reduction in purchase price of components and materials by them.

Next, study estimated Eq 6 by dividing the sample into two subsamples based on firm leverage level, which is measured by firms’ debt to assets ratio. The high leverage (low leverage) contains firms in industries where their debt to assets ratio is greater (smaller) than the median value. Model 2 and 3 obtain similar patterns when applied on Eq (6) for high and low leveraged firms. The findings of results for high leverage and low leverage firms still hold as of full sample firms and strongly support hypotheses *H1a*, *H1b*, *and H1c*. Conclusively, the findings of results imply that reduction in three cash flow measures (ARTD, ITD, and APTD) relate to significant positive improvements in financial performance of firms at current quarter.

### Effect of cash flow metrics on firm financial performance

In this section, the study investigates how changes in cash flow metrics (CCC and OCC) change firm financial performance. Previous literature suggests that lower the level of CCC and OCC better the firm financial performance. Since with longer duration of cash conversion cycle firms may encounter with shortage of liquidity and firm operation may be affected. Hence, the study assumes that changes in CCC and OCC bring positive improvements in firm financial performance. Present study applied following estimation equations of (GEEs) model for cash flow metrics. The model is stated as:

$$\begin{array}{l} \Delta Yit=\beta 0+\beta 1\;\left(\Delta X1it\right)+\beta 2\;\left(\Delta X1it-1\right)+\beta 3\;\left(\Delta X1it-2\right)+\beta 4\;\left(\Delta X1it-3\right)+\beta 5\;\left(\Delta X1it-4\right)+\beta 6\;\left(\Delta X2it\right)+\\ \beta 7\;\left(\Delta X2it-1\right)+\beta 8\;\left(\Delta X2it-2\right)+\beta 9\;\left(\Delta X2it-3\right)+\beta 10\;\left(\Delta X2it-4\right)+\Sigma \beta it\;CONTROLSit+Uit \end{array}$$ (7)

Where ΔY_it_ represents Tobin’s-q for industry i and time t. The ΔX_it_ is ΔCCC and from ΔX_1it-1_ to ΔX_1it-4_ are lags for ΔCCC. The ΔX_2it_ is OCC and from ΔX_2it-1_ to ΔX_2it-4_ are lags for ΔOCC. The CONTROLS_it_ shows the control variables; Size and ROA. The U_it_ is probabilistic term. Study includes four lag effects in Eq 7 for cash flow metrics to examine how long the impact of changes in CCC and OCC on changes in firm performance persists.

Table 4 represents results for cash flow metrics (CCC and OCC). H2a and H2b predict that changes in ΔCCC and ΔOCC bring positive changes in the firm financial performance. The coefficient for the cash conversion cycle (ΔCCC) is -0.0382176, which is statistically significant at a 5% confidence level in the current quarter (as shown in Table 4 column 2).

**Table 4:** Impact of cash flow metrics on firm performance.

Ind. Variables	Full Sample Firms Model 1	High leverage Firms Model 2	Low leverage Firms Model 3
Constant	42.36469*** (8.056291)	42.67042*** (8.068089)	44.43621*** (8.136062)
ΔCCC_t_	-0.0382176* (0.0165285)	-0.4038345** (0.1722255)	-0.027272*** (0.0041239)
ΔCCC_t-1_	0.0001014*** (6.23e-06)	0.0001032*** (4.98e-06)	-0.0045377 (0.006965)
ΔCCC_t-2_	0.0001231*** (0.0000182)	0.0001574*** (0.0000185)	-0.011484 (0.0287961)
ΔCCC_t-3_	-0.0006579*** (0.0000185)	-0.0006571*** (7.29e-06)	0.0056967 (0.0095839)
ΔCCC_t-4_	-0.0003353*** (0.000015)	-0.0003612*** (0.0000104)	0.0048754 (0.0042735)
ΔOCC_t_	0.0039801 (0.0064113)	-0.0572725** (0.0217096)	0.006756 (0.0037125)
ΔOCC_t-1_	0.0002324*** (6.89e-06)	0.0002286*** (4.20e-06)	0.0062759 (0.0065223)
ΔOCC_t-2_	1.91e-06 (0.0000111)	1.05e-06 (9.85e-06)	0.0006349 (0.0051006)
ΔOCC_t-3_	-0.0000446** (0.0000144)	-0.0000404*** (0.0000105)	-0.0070088* (0.0036703)
ΔOCC_t-4_	-0.0000141 (0.0000144)	-7.78e-06 (8.67e-06)	-0.0198116 (0.0115328)
Size	-2.004471*** (0.3939072)	-2.024939*** (0.3946012)	-2.09831*** (0.3972876)
ROA	-0.3909683*** (0.0781972)	-0.3797323*** (0.0782983)	-0.3757659*** (0.0782602)
Wald χ^2^ Statistic	37095.26	396393.08	464.17
No of groups	2536	2536	2536

Note: Standard errors are in parentheses.

***, **, *; indicate significant at 0.1%, 1%, and 5% respectively.

Please check S1 Appendix for variables descriptions.

Next, the study estimated Eq 7 by dividing the sample into two subsamples based on firm leverage level which is measured by firms’ debt to assets ratio. The results in Table 4 Column 3 posit findings for highly leveraged firms. The coefficient for ΔCCC is -0.4038345, which is statistically significant at a 1% confidence level in the current quarter, as shown in Table 4 Column 3. The coefficient for ΔOCC is -0.0572725, which is statistically significant at a 1% confidence level in the current quarter, as shown in Table 4 Column 3. The coefficient for ΔCCC is -0.027272, which is statistically significant at a 0.1% confidence level, as shown in Table 4 column 4 for low-leverage firms at the current quarter.

As predicted by the hypothesis *H2a*; the findings of results also show significant negative association of CCC with firm financial performance at current quarter for full sample firms, high leveraged firms, and low leveraged firms. These evidences of results are consistent with existing literature and show that decline in cash conversion cycle brings positive improvements in firm financial performance [13, 23, 75, 76, 96, 97, 119]. A study by Zeidan and Shapir [24] finds that reducing the CCC by not affecting the sales and operating margin increases the prices of shares, profits, and free cash flow to equity. Moreover, Prior research view that careful handling of the cash conversion cycle leads firms to significantly higher returns [13, 23, 75, 76, 97]. This outcome is consistent with the research by Simon et al. [120], Soukhakian and Khodakarami [121], Basyith et al. [6], Yousaf et al. [60], and Bashir and Regupathi [2]. The findings of the results show a significant negative association of OCC with firm financial performance in the current quarter for highly leveraged firms. The findings suggest that change in OCC led to changes in corporate performance provides significant support to the use of OCC as an indicator for managers to monitor performance and as a lever to manipulate to improve the corporate financial performance. The findings show that OCC in the current quarter posits a significant negative relationship with firm financial performance for highly leveraged firms. This evidence is consistent with the empirical findings of Churchill and Mullins [26].

### Difference of coefficients across high leverage and low leverage firms

In addition, in the next section the present study analyzed the difference of coefficients across two groups by dividing sample into two subsamples, high leveraged and low leveraged firms based on their total debt to total assets ratios. In order to check the difference of coefficients across two groups study applied seemingly unrelated regression (SUR) system on Eqs (6) and (7) to better isolate the effect of cash flow measures and metrics on firm financial performance. The study computed standard errors for differenced coefficients via the seemingly unrelated regression (SUR) system that combines two groups.

The Table 5 reports results for differential impact of cash flow measures and metrics on firm performance across high leverage and low leverage industries. The study finds that the estimated coefficients for differences are positive and statistically significant. These findings of results imply that low leveraged industries are better off in terms of changes in cash flow measures and metrics that bring more positive changes in low debt industries financial performance. Since, low cash conversion cycle (CCC) conserves the debt capacity of the firm as in this situation firms need less short term borrowing to provide liquidity [97]. Therefore, lower cash conversion cycle (CCC) lessens the requirement for lines of credit and contributes to the firms’ debt capacity [23]. Due to high financial distress and higher likelihood of bankruptcy high leverage firms are more bounded by financial constraints which may hinder them to take valuable investments and, thus, harm their profitability [122]. This also suggests that firms with low leverage are high value firms and maintain lower duration of cash conversion cycle (CCC) at low levels that counts to higher profitability which ultimately leads to higher retained earnings and reduce the need for debt.

**Table 5:** Difference of coefficients across high leverage and low leverage firms: Seemingly unrelated regression (SUR) estimation results.

Variables	High leverage minus low leverage firms			
	Coefficients	Standard error	Z-value	P-values
ΔARTD	0.0002917	0.0002526	1.15	0.248
ΔITD	0.001108***	0.0000848	13.06	0.000
ΔAPTD	0.0067759***	0.0002681	25.27	0.000
ΔCCC	0.0004345***	0.0000414	10.51	0.000
ΔOCC	0.0008687***	0.0000715	12.15	0.000

Note:

***, **, *; indicate significant at 1%, 5%, and 10% respectively.

Please check S1 Appendix for variables descriptions.

### Test of endogeneity effect and sensitivity analysis

To further identify an endogeneity concern in estimated results following Arellano and Bover [28] and Blundell and Bond [29] study uses system GMM estimator to alleviate the endogeneity to further investigate the effect of changes in cash flow measures and metrics on firm financial performance. The system GMM method of estimation provides consistent parameter estimates by utilizing instruments that can be obtained from the orthogonality conditions that exist between the lagged values of the variables and disturbances [123]. The system GMM model controls unobserved heterogeneity and potential problems of endogeneity which cash literature has often highlighted [124]. The significant benefit of using GMM estimator is that GMM estimator is robust to capture endogeneity issues and also controls serial correlation problem. Precisely, the present study following Blundell and Bond [29] measured two estimators that can expand the accuracy of the standard first-differenced GMM estimator for the GMM models of this paper. One method enforces an added constraint on the primary settings process, under which all the moment conditions accessible can be exploited by a linear GMM estimator in a system of first-differenced and levels equations. The second method situations, on the pragmatic early standards, to gain a system that under certain conditions can be projected constantly by error components, known as generalized least square (GLS). The finite sample properties of these estimators were studied using Monte Carlo simulations. Both can increase vibrantly on the performance of the usual first-differenced GMM estimator when the autoregressive parameter is moderately high and the number of time-series observations are moderately small. Besides, asymptotic variance comparisons recommend that the system GMM estimator can be significantly more effectual than non-linear GMM in this case. Our results extend certainly towards dynamic models with regressors. The AR (2) test represents the test for residual’s second-order serial correlation in the differenced equation, asymptotically distributed as (0, N) under the hypothesis of no serial correlation. The study follows Monte Carlo simulation where in Eq (8), *i* = 1, 2, …,N and *t* = 2, 3, …, *T*, wherein each case the *Ƞi* and *ɛ*_*i*,*t*_ are drawn as mutually independent i.i.d. N(0,1) [29]. Moreover, following Blundell and Bond’s [29] study employed option robust to obtain robust standard errors after levels equations in all system GMM estimations. Sargan test represents the test for over-identifying restriction asymptotically distributed as chi-square under the null of instrument validity. The study employs following GMM equations:

$$\Delta Yit\;=\beta 0+\beta 1\;\left(\Delta Yit-1\right)+\Sigma \;\beta it\left(\Delta Xit\right)+\Sigma \;\beta it\;CONTROLSit+\lambda t\;+\;Ƞi\;+\;ɛit$$ (8)

Where ΔY_it_ represents firm performance, ΔY_it-1_ is first lag of dependent variable firm performance. All the independent variables (cash flow measures and metrics) are denoted with ΔX_it_. CONTROLS_it_ represents control variables and λ_t_ shows time fixed effects, Ƞ_i_ represents industry fixed effects, and ɛ_it_ represents unobserved heterogeneity factors.

Table 6 represents estimated results obtained using Eq (8). The findings of study observes significant negative association between cash flow measures, metrics and firm financial performance in the full sample, high leverage and low leverage subsamples, indicating that firms’ changes in cash flow measures and metrics bring significant positive improvements in financial performance. Overall, the results still hold after study considers the endogeneity problem, supporting the hypotheses of the study.

**Table 6:** Impact of cash flow measures and metrics on firm performance: GMM estimation.

Variables and Statistics	Cash flow measures			Cash flow metrics		
	Full Sample Firms	High Leverage Firms	Low Leverage Firms	Full Sample Firms	High Leverage Firms	Low Leverage Firms
Constant	-33.13245 ** (-2.24)	145.5175*** (6.80)	-220.795*** (-4.93)	240.3589** (2.48)	566.6316*** (3.23)	311.1894*** (4.03)
ΔARTD	-0.0042108 ** (-2.33)	-0.0000167*** (-2.90)	-2.945264*** (-3.26)			
ΔITD	-0.0004095** (-1.98)	-2.589179*** (-4.83)	-0.0018355*** (-4.87)			
ΔAPTD	-0.0417747* (1.94)	-0.1300243** (1.98)	-0.8954899*** (-4.73)			
ΔCCC				-0.0648145*** (-4.70)	-0.1611204*** (-5.33)	-0.1409594*** (-2.82)
ΔOCC				-0.7870554* (-1.94)	-0.5915164*** (-3.65)	-1.386398*** (-4.60)
Size	-0.0670225 (-0.10)	-6.815052*** (-6.63)	9.192379*** (4.38)	-12.54353*** (-2.62)	-29.25983*** (-3.23)	-15.51277*** (-4.03)
ROA	-0.7732236*** (-3.34)	-1.927879*** (-4.48)	2.82099*** (4.45)	3.805457* (1.68)	3.503284* (1.95)	3.294741* (1.80)
Time fixed effects	Yes	Yes	Yes	Yes	Yes	Yes
Industry fixed effects	Yes	Yes	Yes	Yes	Yes	Yes
AR(_2_)—P-value	0.075	0.563	0.621	0.082	0.283	0.232
Sargan Test-P-value	0.999	0.951	0.501	0.891	0.999	0.457
No. of groups	2536	2536	2536	2535	2536	2536
Observations	9478	9478	9476	9381	9431	9473

Note: The t-statistics in brackets.

***, **, *; indicate significant at 1%, 5%, and 10% respectively.

Please check S1 Appendix for variables descriptions.

In addition, the study further employs a sensitivity analysis specifications to better isolate the impact of changes in cash flow measures, metrics on firm financial performance. Since changes in cash flow measures and metrics may also affect the firm’s financial performance in previous period. In order to investigate influence of changes in cash flow measures and metrics on firm performance in previous period the present study replaced main independent variables with their one period lagged variables. The study regresses firm performance on cash flow measures and metrics with other potential determinants as follows:

$$\Delta Yit=\beta 0+\Sigma \;\beta it\left(\Delta Xit-1\right)+\Sigma \;\beta it\;CONTROLSit+Dt+Di+ɛit$$ (9)

Where ΔY_it_ represents firm performance. All the independent variables (cash flow measures and metrics) are denoted with ΔX_it-1_, and CONTROLS_it_ represents control variables. D_t_ shows time fixed effect, D_i_ represents industry fixed effects, and ɛ_it_ represents unobserved heterogeneity factors.

Table 7 represents estimated results of sensitivity analysis regression. The study finds that estimated coefficients of cash flow measures (ΔARTD_t-1_, ΔITD_t-1_, ΔAPTD_t-1_) and cash flow metrics (ΔCCC_t-1_, ΔOCC_t-1_) are negative and significant, indicating that changes in previous period’s cash flow measures (ΔARTD_t-1_, ΔITD_t-1_, ΔAPTD_t-1_) and cash flow metrics (ΔCCC_t-1_, ΔOCC_t-1_) bring significant positive changes in firm financial performance. The study finds similar results to the previously reported findings for alternative subsamples of high leverage and low leverage firms. Overall, the sensitivity analysis results still hold in consistent with the primary analysis results and ensure robustness of main analysis results of the study.

**Table 7:** Impact of cash flow measures and metrics on firm performance: Sensitivity analysis.

Variables and Statistics	Cash flow measures			Cash flow metrics		
	Full Sample Firms	High Leverage Firms	Low Leverage Firms	Full Sample Firms	High Leverage Firms	Low Leverage Firms
Constant	28.56149*** (67.37)	25.98236*** (66.03)	21.62801*** (35.22)	35.35247*** (22.86)	24.32444*** (63.91)	25.81588*** (65.14)
ΔARTD_**t-1**_	-0.0006093* (-1.85)	-0.0008755* (-1.82)	-0.0023337** (-2.10)			
ΔITD_**t-1**_	-0.0014659*** (-13.43)	-0.0014707*** (-10.23)	-0.0007248*** (-2.64)			
ΔAPTD_**t-1**_	-0.0012615*** (-3.28)	-0.0009463* (-1.80)	-0.0019635* (-1.81)			
ΔCCC_**t-1**_				-40.82176*** (-5.91)	-1.985654*** (-7.43)	-0.0005828** (-2.52)
ΔOCC_**t-1**_				-6.13e-06 (-1.00)	-0.000012** (-2.08)	-0.0001533*** (-3.35)
Size	-1.280058*** (-63.59)	-1.104578*** (-57.80)	-0.9262797*** (-31.73)	-1.165941*** (-46.04)	-1.014402*** (-55.62)	-1.085568*** (-60.02)
ROA	0.1751111*** (18.78)	0.101579*** (13.48)	0.2058251*** (25.83)	0.1504932*** (20.06)	0.180005*** (18.32)	0.1467727*** (21.87)
Time fixed effects	Yes	Yes	Yes	Yes	Yes	Yes
Industry fixed effects	Yes	Yes	Yes	Yes	Yes	Yes
R-Squared	0.2419	0.2227	0.1954	0.2206	0.2388	0.2356
F-Stat	446.24***	684.05***	153.13***	333.19***	375.02***	397.89***
Observations	16799	14335	9476	9426	16748	16799

Note: The t-statistics in brackets.

***, **, *; indicate significant at 1%, 5%, and 10% respectively.

Please check S1 Appendix for variables descriptions.

### Practical, managerial, and regulatory implications

This study provides significant practical, managerial, and regulatory implications for cash flow management and working capital management decisions in the corporate sector to improve performance. Most studies on cash flow management have focused on its relationship to profitability from the perspective of manufacturing companies. This research focuses on cash flow management by linking the leverage of non-financial firms in the Chinese context, a fundamental issue of corporate cash flow management and working capital investment that has not been studied much in the emerging markets scenario. Practically study suggests that a decline in cash flow measures and metrics positively enhances a company’s financial performance. Moreover, the paper determines that low-leverage industries perform healthier to cash flow measures and metrics changes. The study also reveals that companies in low-debt industries experience more positive improvements in their financial performance relative to high-debt industry companies. Therefore, the findings of this paper suggest that highly leveraged companies may be less conducive to improving corporate performance in industries where competitors’ leverage is relatively low.

Thus, from managers’ and policymakers’ points of view, the analysis found that changes in cash flow measures (ARTD, ITD, and APTD) and metrics (CCC and OCC) have led to significant positive improvements in the company’s financial performance. These positive changes in the CCC mean that changes in the accounts payable cycle appear to mitigate the combined impact of changes in the accounts receivable and inventory cycles. For managers, this finding suggests that reducing CCC simply by lowering APTD can translate into improvements in company performance. These findings provide rich insights and practical implications for managers and policymakers to use CCC as an operational tool to improve company performance. Therefore, managers and policymakers must actively evaluate the company’s policies regarding cash flow management, working capital management, corporate leverage, and capital budgeting policy before capitalizing on these companies.

## Conclusion, limitations, and future implications

### Conclusion

Cash flow management is the central issue of company operational strategies that affect a firm’s operational decisions and financial position. Firms’ effective policy of cash flow management is achievable through efficient management of working capital, which is possible through shorter days of accounts receivables, giving discounts on prompt payments, offering cash incentives, reducing inventory turning days through sound inventory management policies, shortening days of accounts payable by achieving rebate on early outlays. Likewise, inventory turnover may lead to a significant positive relationship with organizational performance symbolized by return on assets, cash flow margins, and return on sales in the JIT context. Moreover, high-performance firms may have a lengthier duration of days of accounts payables, which ensures the presence of liquidity. Many firms invest a large portion of their cash in working capital, which suggests that efficient working capital management significantly impacts corporate profitability.

This paper offers a strong insight and findings on cash flow management and firm financial performance by examining the Chinese full sample firms, high debt, and low debt firms to investigate the impact of changes in cash flow measures and metrics on firm performance. Using the exclusive cash flow measures and metrics data, study finds that decline in cash flow measures and metrics bring significant positive changes in firm financial performance. Moreover, study finds that low leveraged industries are better off in terms of changes in cash flow measures and metrics that bring more positive improvements in low debt industries firms’ financial performance relatively to high debt industries firms. This paper also demonstrates that, following firms’ leverage, high-leveraged firms may be less advantageous to enhance firm performance in industries where rivals are relatively low-leveraged.

The results of the study are consistent with the argument that changes in cash flow measure (ARTD, ITD and APTD) and metrics (CCC and OCC) bring significant positive improvements in firm financial performance. These findings furnish a great amount of insight and practical implication for manager to utilize CCC as operating tool in order to enhance firm performance. Firms by actively monitoring and controlling levers such as; ARTD, ITD, APTD, CCC, OCC can enhance financial performance. The findings of results are robust to different measures and metrics of cash flow and firm financial performance, following sensitivity analysis and endogeneity test still main results hold and ensures the robustness of primary analysis.

### Study limitations and directions for future research

This research is of great significance to the studies on the relationship between cash flow management and enterprise performance in the Chinese market environment. However, the study did not consider some aspects that need consideration in future studies. This study uses Tobin Q to measure a company’s performance. However, it is also possible to include other company performance indicators that are important in the strategic impact of studies and may provide significant insights. The lack of data availability is a major constraint due to companies’ exits and entry into the sample period. This paper uses secondary data; however, studies can also use primary data to understand and gain appropriate knowledge of corporate cash flow management by combining archived and survey data to improve the robustness and significance of research findings in the context of emerging markets. This study focuses on the financial performance of firms. However, future studies can also use non-financial performance as a consequence variable.

Future extensions of this work may examine whether a company’s cash flow management policies in other areas of the supply chain have a similar relationship to company performance.

In addition, further inquiries that explore the directional association amid inventory and performance changes may extend the understanding of the cash flow management role in a company’s success. In addition, there is a need to explore more the impact of cash flow and working capital investment on firm performance by taking the market imperfections within the framework of emerging economies. Finally, the evidence of this research from the fastest emergent economy of the world may also use other transition economies to generalize for a widespread population group. Finally, studies in the future can consider linking product market competition with the cash flow measures, metrics, and firm performance relationship.

## Supporting information

S1 Appendix (PDF)
