# Supplementary material for: Cash flow management and its effect on firm performance: Empirical evidence on non-financial firms of China
Source: PLoS One. 2023 Jun 20;18(6):e0287135. doi: 10.1371/journal.pone.0287135 (PMC10281586; doi:10.1371/journal.pone.0287135)
Supplement: S1 Appendix — (PDF) [file pone.0287135.s001.pdf]

Appendix 1: Description of Variables

Variables	Abbreviation	Measurement
**Dependent Variable**		
Firm Performance	Tobin's-q	(Equity value + Book value of long-term debt + Net current liabilities)/ (Value of total assets) [1, 103, 104, 105, 106].
**Independent Variables**		
Accounts Receivables Turning Days	ARTD	(Accounts receivable/sales)* No. of days in a period [5, 12].
Inventory Turning Days	ITD	(Inventory/cost of goods sold)* No. of days in a period [1, 12].
Accounts payable Turning Days	APTD	(Accounts payable/purchases)* No. of days in a period [12, 76].
Operating Cash Cycle	OCC	Accounts payable turning days + Inventory turning days [1, 26].
Cash Conversion Cycle	CCC	Accounts payable turning days + Inventory turning days - Accounts payable turning days [1, 76, 97].
**Control variables**		
Size	Size	Natural logarithm of quarterly sales [76].
Return on Assets	ROA	EBIT / Total assets [96, 110].
Industry Dummy	Industry Fixed Effects	Dummies of industries to control potential influence of industries.
Time Dummy	Time Fixed Effects	Dummies of quarters to control the effect of time.
